# Driver’s Personality and Behavior for Boosting Automobile Security and Sensing Health Problems Through Fuzzy Signal Detection Case Study: Mexico City

**DOI:** 10.3390/s21217350

**Published:** 2021-11-05

**Authors:** Germán E. Baltazar Reyes, Pedro Ponce, Sergio Castellanos, José Alberto Galván Hernández, Uriel Sierra Cruz, Troy MacDaniel, Arturo Molina

**Affiliations:** 1 School of Engineering and Sciences, Tecnologico de Monterrey National Department of Research, Mexico City 14380, Mexico; A01331329@itesm.mx (G.E.B.R.); josegalvan_94@hotmail.com (J.A.G.H.); A01171504@itesm.mx (U.S.C.); armolina@tec.mx (A.M.); 2 Civil, Architectural and Environmental Engineering, Cockrell School of Engineering, University of Texas at Austin, Austin, TX 78712, USA; sergioc@utexas.edu; 3 Center for Cognitive Ubiquitous Computing (CUbiC), Arizona State University, Tempe, AZ 85281, USA; troy.mcdaniel@asu.edu

**Keywords:** signal detection, fuzzy logic, personality, health problems

## Abstract

Automobile security became an essential theme over the last years, and some automakers invested much money for collision avoidance systems, but personalization of their driving systems based on the user’s behavior was not explored in detail. Furthermore, efficiency gains could be had with tailored systems. In Mexico, 80% of automobile accidents are caused by human beings; the remaining 20% are related to other issues such as mechanical problems. Thus, 80% represents a significant opportunity to improve safety and explore driving efficiency gains. Moreover, when driving aggressively, it could be connected with mental health as a post-traumatic stress disorder. This paper proposes a Tailored Collision Mitigation Braking System, which evaluates the driver’s personality driving treats through signal detection theory to create a cognitive map that understands the driving personality of the driver. In this way, aggressive driving can be detected; the system is then trained to recognize the personality trait of the driver and select the appropriate stimuli to achieve the optimal driving output. As a result, when aggressive driving is detected continuously, an automatic alert could be sent to the health specialists regarding particular risky behavior linked with mental problems or drug consumption. Thus, the driving profile test could also be used as a detector for health problems.

## 1. Introduction

Early automotive warning systems were implemented since the late 1950s to increase vehicle safety [1]. One of the first prototypes created was the Cadillac Cyclone [1], which used radar technology to detect objects in front of the car. In 1995, a research team of Hughes Research Laboratories (HRL) developed the Forewarn, a radar-based forward collision avoidance system [2]. Unfortunately, these models’ expensive manufacturing costs prevented their mass production and marketing.

At the beginning of the 2000s, several investigations were published regarding the viability and usability of frontal collision warning systems. For example, the Insurance Institute for Highway Safety [3] discovered that the use of autonomous avoidance and adaptive headlights regarding the driver’s steers considerably reduced insurance coverage in car accidents. However, the U.S. National Highway Traffic Safety Administration (NHTSA) considered that it is still not mandatory the usage of these systems in commercial vehicles [4].

Besides, research shows how mental health problems or drug consumption can be linked with persons who drive aggressively [5,6]. According to Lashkov and Kashevnik [7], aggressive driving refers to a driving behavior that may create a dangerous situation for the driver and other people on the road. In that sense, the driving profile could be seen as a detector of mental health problems. Also, the acceleration pedal force and the speed relative to the speed limit can be added as a sensor for detecting an aggressive driving condition.

Current systems are noncustomizable: they only emit a fixed visual and audible alert and do not consider the driver’s personality, nor which stimuli are better for the driver’s response and engagement. This work analyzes the individual driving personality of the user through signal detection theory to generate a cognitive map that evaluates and classifies the general driving behavior of a person. The most compatible stimuli are selected according to their behavior to react to aggressive behavior while driving and prevent a possible accident.

This paper is developed as follows: Section 2 summarizes the state-of-the-art in collision avoidance systems and the different approaches to analyzing drivers’ personalities, as well as a general description of signal detection theory (SDT). Section 3 introduces our contribution to determine the driver’s personality. Section 4 shows the results and their corresponding analysis. Finally, Section 5 highlights the respective conclusions of this work.

## 2. Background

### 2.1. Collision Avoidance Systems

In the context of Mexico, its Federal Police [8] indicate that the causes of accidents on federal roads, about 80% of the time, are due to the drivers’ negligence, 7% vehicle malfunction, 9% to natural hazards, and only 4% to road and road conditions.

Mexican Federal Police [8] reports and statistics indicate that human factors cause the highest percentage of traffic accidents in Mexico. The leading causes include driving under the influence of alcohol, medicines, and narcotics, perform risk maneuvers, driving at excessive speed, driving with fatigue, tiredness or sleepiness, and the driver’s physical health.

The next factor is mechanical: it includes a vehicle in unsuitable conditions for operation and improper maintenance. The third place is the climatic factor, which includes: fog, humidity, landslides, unstable areas, and subsidence. Finally, there are structural transit factors, including road marking errors, roads in poor conditions, and the lack of paint and reflectors in the central and lateral lines of the road.

Automatic emergency braking (AEB) systems detect an impending forward crash with another vehicle in time to avoid or mitigate the crash [9]. These systems first alert the driver to take corrective action to avoid the crash. If the driver’s response is not enough to avoid the crash, the AEB system may automatically apply the brakes to prevent or reduce the severity of a crash. AEB systems have the potential to save lives and reduce moderate and less severe rear-end crashes [9] that are common on our roadways. A 2015 study based on European and Australasian data suggests the AEB can decrease rear-end collisions by 38% [10] using a radar-based distance measuring device that alerts the driver if a collision is imminent.

AEB systems warn the driver every time a possible accident (collision) may occur. In the case that the system detects that the accident is unavoidable, it takes control of the vehicle’s behavior instead of the driver; under this situation, the AEB system is capable of braking, emitting a sound, create a visual alert, or steering autonomously [11]. According to [12], different actions are necessary according to the situation: collision avoidance systems are appropriate for low vehicle speeds, while collision avoidance is preferred at higher vehicle speeds (considering if lanes are empty).

These systems alert the driver visually and audibly. However, these alerts could be ignored when a driver is associated with post-traumatic stress disorder, traumatic brain injury, or drug consumption [5,6]. Thus, there are research papers that proposed several alternatives for detecting aggressive driving [13,14,15]. Furthermore, the proposed methodology presented in this paper integrates differently the detection of aggressive driving and the possible actions that have to be taken to decrement automotive accidents and detect health problems.

This paper seeks better stimuli that can alert the driver faster and safer. Besides, if this aggressive driving condition continues, an automatic alarm can be sent to the health experts. The project is looking for a braking and alert system which could be personalized using speed and distance variables: the Tailored Collision Mitigation Braking System (TCMBS) could alert the driver if a collision is imminent, adjust the seatbelt tension, and partially or fully apply the brakes depending on the variables. Moreover, the acceleration pedal force and the speed relative to the speed limit could be part of the sensor system for detecting aggressive driving conditions when it is necessary.

**Figure 1:**
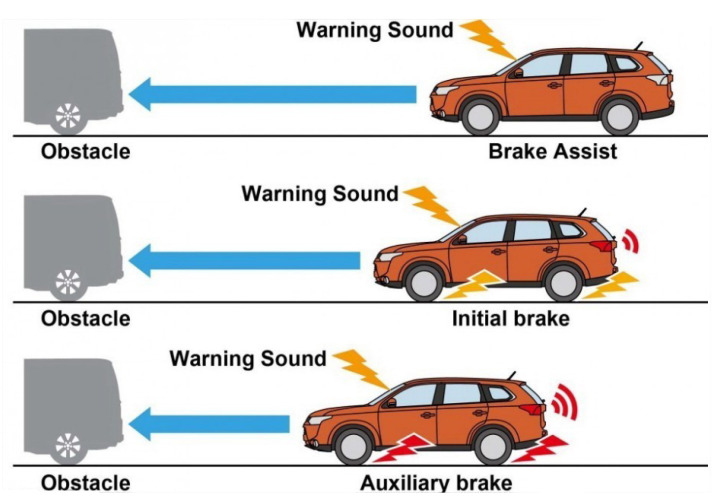
Basic CMBS system description.

One example of these systems is Honda’s Collision Mitigation Braking System (CMBS) [16]. It monitors the distance between a car and the car at the front. If it determines that the distance is reduced, it displays a “BRAKE” message on display, and the steering wheel vibrates. If the driver does not react, the system lightly retracts the seatbelt and applies a little braking force. In the possible outcome that the driver does not act, the system determines that the collision is unavoidable and applies a stronger seatbelt retraction and braking force to reduce the severity of the impact. Figure 1 shows how the CMBS reacts under possible collision circumstances.

Nevertheless, the proposed system in this paper detects a profile of the driver previously so a prior knowledge can be comprised into the response system. When aggressive or risky driving remains, an alert could be sent to the health experts. As a result, it could be determined if the driver is associated with mental health problems.

### 2.2. Driver’s Personality Analysis

Even though the development of systems capable of preventing or dealing with possible automobile collisions is not a new research area, it is still required to analyze how the car needs to behave during these situations and evaluate the factors that influence the driver to reach this accident situation. Although most studies and intuitive assumptions suggest that road traffic accidents are directly related to the driver’s skill and ability, it was recently noticed that solely skill is not enough to ensure safe driving [17].

The driver’s personality traits analysis is an important research area that evaluates the person’s tendencies under particular stressful situations when driving, rather than evaluating only driving skills and experience. The work presented in [17] demonstrates no considerable difference between risky and non-risky drivers driving abilities; however, the same study found that the analysis of personality traits helps predict safe or risky driving behaviors.

Zicat et al. [18] evaluated the relationship between the attitudes and personalities of people when driving. Their study analyzed the driver’s attitudes towards road safety, correlated the driver’s personality based on their anxiety, anger, and sensation-seeking attitudes under particular circumstances, and compared them to the results of different cognitive tests. Their conclusions found that personality traits and cognitive skills are highly related to driving behavior, especially with younger drivers.

Another study that compared personality traits with risky driving and crash possibilities among newly licensed drivers found that the crashing rate of a driver was directly related to their personality traits. Nevertheless, the study also determined that the correlation between these two variables was much smaller on new drivers since inexperience outweighs their personality influence when driving during the first months [19].

Finally, the work presented by Jonsson and Dahlbäck [20] investigated how an aggressive or submissive match between the driver and an in-vehicle system affects the driving behavior of the user. In this case, the voice traits of the driving system affected the driver’s reactions. This study concluded that the match between the personalities of the system and the driver improved the driving performance of the latter, even though the statistics showed that every user preferred to be guided by an aggressive system.

Even though most of the studies previously mentioned evaluated the user’s personality traits, it is still necessary to evaluate the certainty of the user when responding to their evaluations and determine under which particular situations the same user can change from a calm driver to an aggressive one. At the same time, it is also required to compare the results of the analyzed population with an expert that can determine the ideal way of how their reactions under certain situations should be.

### 2.3. Signal Detection Theory

Signal Detection Theory (SDT) is used to analyze data coming from experiments where the task is to categorize ambiguous stimuli which can be generated either by a known process (called the signal) or be obtained by chance (called the noise in the SDT framework). SDT assumes two possible states of the world: signal (s), in which the event of interest is present, and noise (n), in which it is absent [21]. At any given time, one of these states of the world occurs. The detection system (human or machine, or some combination) makes a yes (Y) or a no (N) judgment, indicating whether or not it is believed that the signal is present or absent [22]. SDT is used in very different domains from psychology (e.g., psychophysics, perception, memory), medical diagnostics (e.g., do the symptoms match a known diagnostic? or can they be dismissed as irrelevant?), and statistical decision (e.g., does the data indicate that the experiment affects or not?) [21].

The proportions of Hits and (False Alarms) FAs reflect the effect of two underlying parameters: the first reflects the separation between the signal and the noise, and the second is the participant’s strategy. The goal of SDT is to estimate the value of these two parameters (Hit and Correct Rejection) from the experimental data.

Across many such occurrences or trials, the two possible states of the world and the two possible decisions result in four possible outcomes, each with an associated probability (P). These states are better described in Equations (1)–(4). A “Yes” response given to an old stimulus is a correct response and is called a Hit, but a “Yes” response to a new stimulus is a mistake and is called a False Alarm (FA). A “No” response given to a new stimulus is a correct response and is called a Correct Rejection (CR), but a “No” response to an old stimulus is a mistake and is called a Miss (M).

(1) H=min(s,r)

(2) M=max(s−r,0)

(3) FA=max(r−s,0)

(4) CR=min(1−s,1−r)

After *n* observations, hit rate (HR), false alarm rate (FAR), miss rate (MR), and correct rejection rate (CRR) are calculated by Equations (5)–(8), respectively [21]:

(5) $$HR=\frac{\sum_{i}^{n}H_{i}}{\sum_{i}^{n}s_{i}}$$

(6) $$FAR=\frac{\sum_{i}^{n}FA_{i}}{\sum_{i}^{n}1-s_{i}}$$

(7) $$MR=\frac{\sum_{i}^{n}M_{i}}{\sum_{i}^{n}s_{i}}$$

(8) $$CRR=\frac{\sum_{i}^{n}CR_{i}}{\sum_{i}^{n}1-s_{i}}$$

Only two of the four probabilities are needed for complete characterization of the performance outcomes since HR+MR=1, and CRR+FAR=1. The convention uses the probability of a Hit or HR and the probability of a FA or FA Rate (FAR) to describe the decision outcomes.

The hit and FA probabilities can then be used to compute various measures of the performance of the detection system. In general, it is necessary to distinguish the sensitivity or bias-free accuracy of the detection system from the criterion or decision threshold associated with the choice of judgments or responses. In SDT, sensitivity is indexed by the parameter $d^{\prime }$ and the criterion by the parameter β [21].

The goal of SDT is to estimate two main parameters from the experimental data. The first parameter, called $d^{\prime }$, indicates the strength of the signal (relative to the noise). The second parameter, called *C* (a variant of it is called β), reflects the strategy of the response of the participant (e.g., saying quickly “Yes” rather than “No”). Both parameters are described by (9) and (10), respectively.

The strategy of the participant is expressed via the choice of the threshold. An alternative way of expressing the position of the participant’s criterion is given by β. It corresponds to the ratio of the height of the signal distribution to the noise distribution for the value of the threshold.

(9) $$d^{\prime }=Z\left(HR\right)-Z\left(FAR\right)$$

(10) $$\beta =e^{\ln(d^{\prime }C)}$$

The criterion location *C* is a measure of response bias. The following expression is used to find its value if the evaluation is related to the point at which the two distributions cross.

(11) $$C=\frac{1}{2}\left[Z(HR)+Z(FAR)\right]$$

The SDT model assumes that the participant’s response depends upon the intensity of a hidden variable (e.g., if the driver always wears a seatbelt while driving) and that the participant responds “Yes” when the value of this variable for the stimulus is more significant than a predefined threshold.

SDT also assumes that the stimuli generated by the noise condition vary naturally for that hidden variable. As is often the case elsewhere, SDT assumes that the hidden variable values for the noise follow a normal distribution. Recall at this point that when a variable *x* follows a Gaussian (a.k.a. Normal) distribution (12), this distribution depends upon two parameters: the mean (denoted μ) and the variance (denoted $\sigma^{2}$). It is defined as:

(12) $$G\left(x,\mu ,\sigma \right)=\frac{1}{\sigma \sqrt{2\pi }}\exp\left\{-\frac{{\left(x-\mu \right)}^{2}}{2\sigma^{2}}\right\}$$

In general, within the SDT framework, the values of μ and σ are arbitrary, and therefore we choose the values of μ=0 and σ=1 (other values will give the same results but with more cumbersome procedures). In this case, (13) reduces to:

(13) $$N\left(x\right)=\frac{1}{\sqrt{2\pi }}\exp\left\{-\frac{1}{2}x^{2}\right\}$$

The standard deviation of the noise is equivalent to the unit of measurement of *x*. The signal distribution is identical to the noise distribution, but it is moved to the right of the noise distribution. The distance between the signal and the noise distribution corresponds to the effect of the signal (this is the quantity added to the noise distribution to get the signal distribution): this distance is called $d^{\prime }$. Because the mean of the noise distribution is zero, $d^{\prime }$ is equal to the mean of the signal distribution. SDT is one of the most relevant procedures to evaluate binary data in a psychological or behavioral test since it can compare the user’s perceptions and decisions under a correct or behavioral test incorrect stimulus. For this reason, the implementation of SDT was widespread when observing particular treats of a given population. For example, Rader et al. [23] used this method to analyze multiple behavioral analysts’ reliability, accuracy, and bias when interpreting data. Their results showed that visual analysis of the evaluated population depended on personal treats, making the overall accuracy of their observations questionable. DeCarlo [24] used SDT to design a multiple-question exam to evaluate how a correct answer is selected among different incorrect (noise) alternatives. Finally, Gruda and Kafetsios [25] compared a patient’s attachment and acceptance of a general practitioner before and after the COVID-19 pandemic and how anxious and avoidant they were when being treated by them.

## 3. Personality Analysis Proposal

The project intends to evaluate different driver personalities based on their driving behavior and assign them a calm or aggressive personality. This analysis is done through the implementation of an SDT-based survey model.

**Figure 2:**
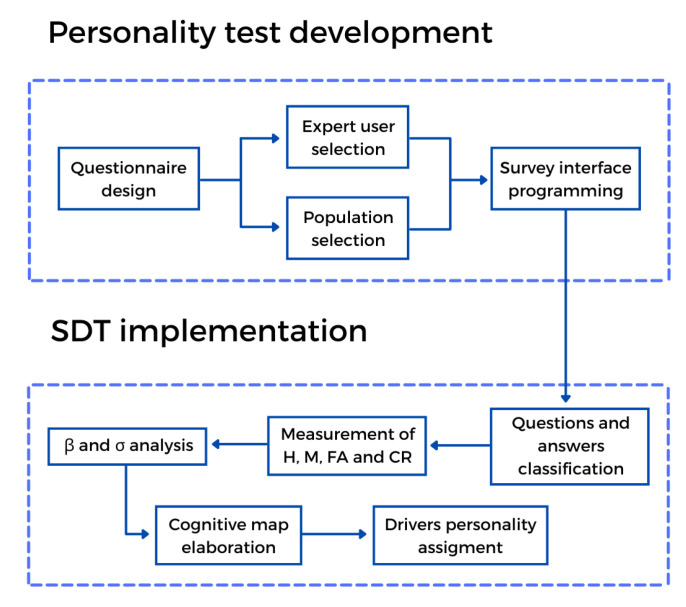
Project flowchart.

To achieve the objectives of this project, it was necessary to implement a driving personality test. Subsequently, the analysis of the test and the results were carried out using the signal detection theory method, which will be the basis for implementing a prototype in Arduino. The progress of the project can be seen in Figure 2.

The test driver personality is determined using a 60-question questionnaire (see Table 1) into two categories: (i) calm or (ii) aggressive driver [26]. The survey includes questions that involved local transit regulations, driving habits, car-based physical phenomes, driving attitudes, and driving responsibility. The test comprises two types of questions: (i) situational and (ii) self-assessment. The survey used for this project has different answers: for questions 1–17, the surveyed person can choose between Always, Often, Sometimes, Rarely, and Never. Questions 18–25 have a binary answer: true or false. Questions 26, 27, 28, 29, and 50–60 involve multiple options answers, shown in Table 2. Finally, for questions 30–49, the person can select between: Quite often, Often, Sometimes, Rarely, and Almost never.

Each surveyed person needs to answer how likely they would react in particular situations. For the self-assessment questions, the person indicates the degree to which the given statements apply to it. The evaluated population was formed by 11 different people who are accustomed to driving every day. The corresponding age range is between 18 and 65 years old, being 50% male and 50% female drivers. The survey was also given to a driving school teacher to obtain an ideal set of answers based on an expert’s point of view about how every answer should be answered. This approach allowed to determine which questions should be classified as a signal present or absent for the SDT implementation.

Two types of driver profiles were considered during this project, intending to obtain more detailed results. The first is considered a good and quiet driver that does not seek to get involved in problems or situations in which their life or their companion’s life is in danger, he/she also respects the traffic regulations, so it can be deducted that he/she respects speed limits and signs. On the other hand, the aggressive driver profile has specific characteristics such as low-tolerance, high-stress, and aggression. With those characteristics, it is adequate to say that there is a driver who quickly loses control of his temper since he/she cannot handle stressful situations that are lived on a daily driving basis, in the same way, their poor tolerance makes them get involved in situations that can put in danger their life, that of their companions or that of other drivers. Finally, a driver with the second profile is more likely to cause a car accident due to their driving.

The questionnaire was programmed into a LabVIEW®interface to allow a smoother interaction with the users, save their corresponding answers directly, and proceed with the SDT analysis of their finished survey. For every question, the LabVIEW®interface showed all the possible answers. Once the questionnaire was completed, the interface compared the user and expert answers to determine the HR and FAR and further measure the β and $d^{\prime }$ values.

Finally, after comparing the SDT results of every user, a cognitive map was designed to evaluate the main characteristics of every one of the driver personalities. These characteristics resulted from analyzing the SDT personality traits of every user and analyzing the collective responses of every question of the survey. This approach allowed the integration of a quantitative and qualitative analysis of the users’ answers.

## 4. Drivers Personality and Behavior Associated with Health Problems

There were multiple investigations regarding the physical and psychological perspectives of aggressive driving. For example, Harro et al. mention that this behavior is mainly caused by a genetic variation of a neuropeptide whose primary role is to maintain wakefulness [27]. Other studies, such as the one presented by Scott-Parker et al. [28], suggest that aggressive driving is only a response to three external situations: the quality and traffic in infrastructures and locations, other drivers, and the stress level the driver has even before entering their car. Some studies evaluate common personality traits of people that may generate traffic fines and accidents regularly [29]. Qi et al. [30] exemplify that a taxi driver compromises their visual fixation points with aggressive behavior, reducing response time when changing lines and possibly producing more accidents.

Other studies correlate aggressive driving with more psychiatric severe disorders, such as intermittent explosive disorder (IED), attention deficit hyperactivity (ADHD) substance use disorders, and antisocial personality [31]. Vanstone et al. [32] even evaluate that uncontrollable situations, such as diabetes, may generate anxiety in a person and influence their driving behaviors. The results presented by van den Berg et al. even demonstrate that aggressive driving is not highly correlated with the driver’s moral values [33], suggesting a more direct physiological reason for this behavior.

Even though there is plenty of information regarding the possible causes behind car accidents and aggressive driving behavior, it is still needed to implement this information into educating, enforcing, and engaging drivers and developing engineering [28] solutions to prevent further accidents. One example is by controlling the use of the seatbelt under aggressive-driver situations [34]. Other authors, such as Dahlen et al. [35], created a model that may predict a crash or a ticket generation based on personality predictors such as agreeableness and driving anger; however, this proposal only evaluates a compound of traffic reports, but no physical implementation was proposed. Dadhich and Gupta [36] present a similar proposal as this paper. These authors propose a device capable of detecting aggressive driving behaviors through the car’s acceleration signature. Unfortunately, this device only detects an acceleration anomaly, but there is no correlation with the driver’s attitude or situation, making it more challenging to determine if the car’s acceleration was a result of aggressive behavior or not.

The value proposal of this paper is that it evaluates, determines, and implements the driver’s behavior into a preventive action on the car. It was demonstrated that by evaluating the personal traits of the driver, it is possible to evaluate if their behavior while driving may cause or end in an accident or not. At the same time, it was also mentioned that it is necessary to link the driver’s personality with the car’s behavior. It is adequate then to determine that using the user’s personality evaluation through SDT allows a quick and practical evaluation of the driver’s behavior while implementing a direct, preventive routine in the vehicle that controls both its acceleration and the stiffness of the vehicle seatbelt. Hence, this proposal combines an evaluation metric with a direct action inside the car that actually may prevent an accident, not just determining if it is going to happen or not. No other reference prevents accidents and evaluates metrics in the same structure. Moreover, the system is a clear indicator for detecting healthy issues by aggressive driving.

## 5. Project Results

### 5.1. Survey Results

After completing the survey, the general statistics of the questionnaire were obtained. Considering that the arithmetic mean of the population was 6 (including both the expert and the regular users), the questions that surpassed that frequency of response determined the general behavior of the population. Questions 1–49 were used for the SDT analysis of the users. Since the final 10 questions were focused on more practical and particular cases, those answers were used to evaluate the different traits of every user to obtain the cognitive map characteristics of every driver’s personality.

**Figure 3:**
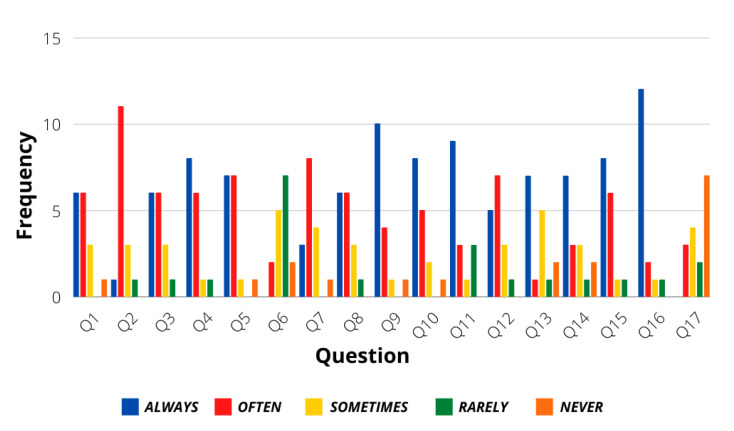
Questions 1–17 results.

**Figure 4:**
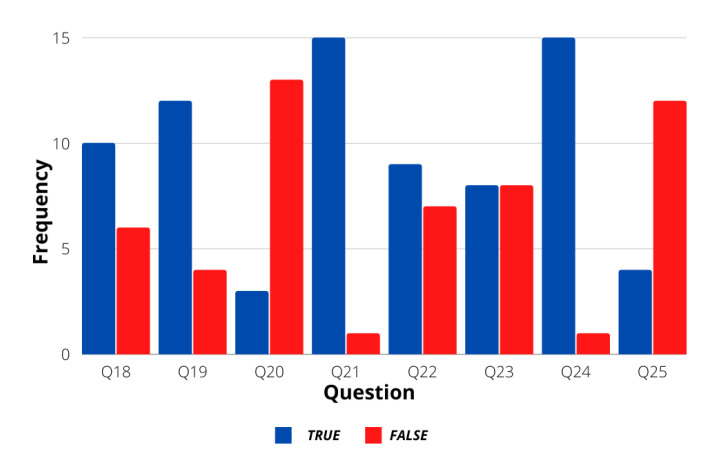
Questions 18–25 results.

**Figure 5:**
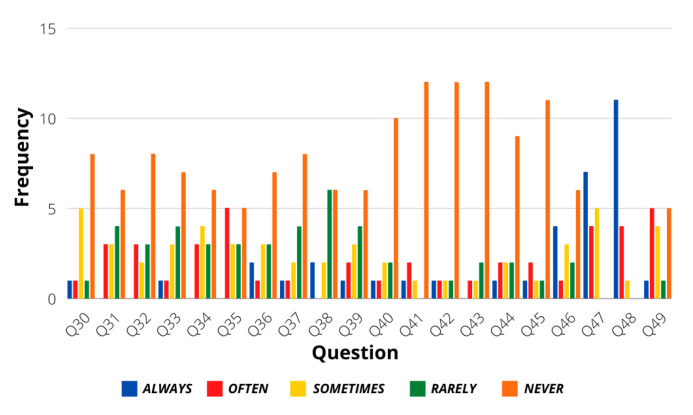
Questions 30–49 results.

Figure 3 shows the statistics of the first group of 5-Likert answers regarding general driving situations. As observed, the general answers of the users followed a calm personality, allowing the assumption that most drivers make use of proper skills when driving under different situations. Figure 4 shows the statistics of the questions that were answered with binary responses. Even though most of the answers in this group also followed a calm personality, the users present a slightly aggressive response when dealing with traffic regulation policies, like in the case of question 18, where they affirm that the first served policy is the best option when reaching an intersection. Finally, another evaluation of calm or aggressive treats was evaluated from questions 30–49, as seen in Figure 5. Again, a calm driver personality is frequently observed in the population.

**Figure 6:**
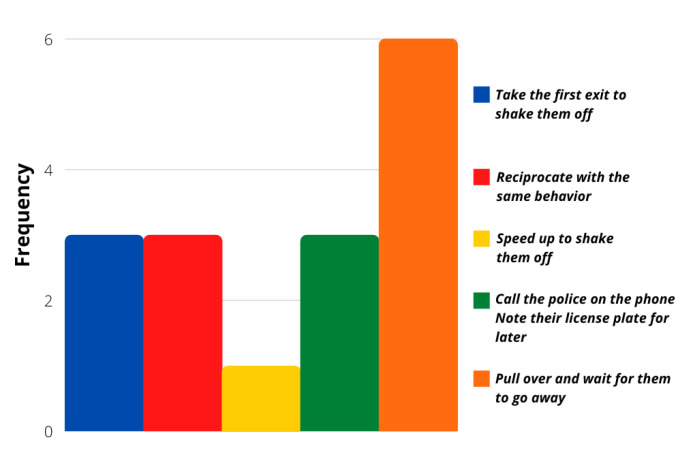
Question 50 results.

**Figure 7:**
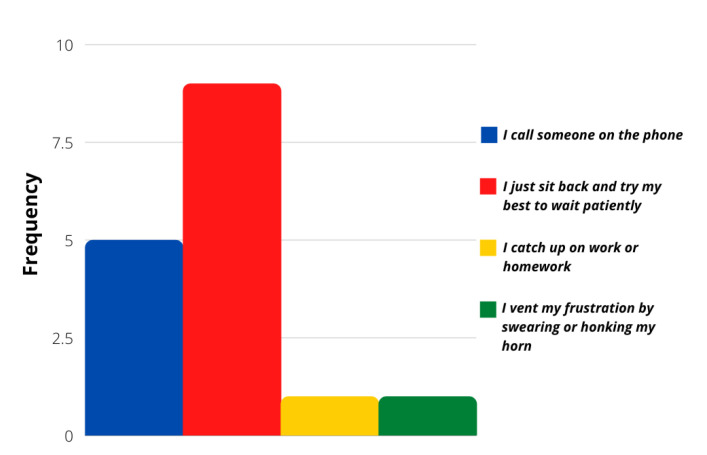
Question 51 results.

**Figure 8:**
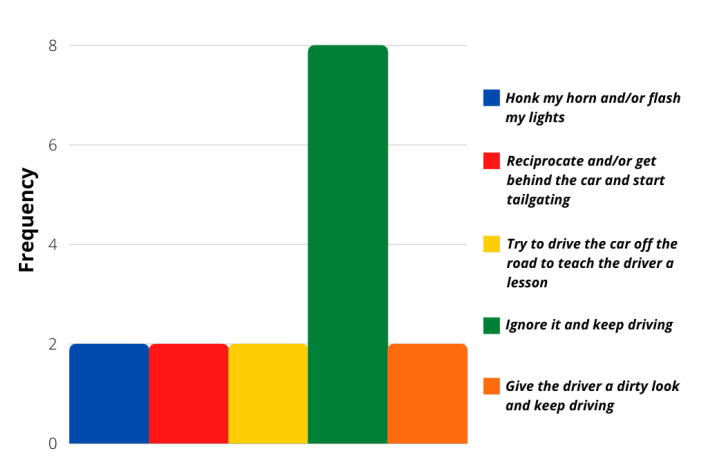
Question 54 results.

**Figure 9:**
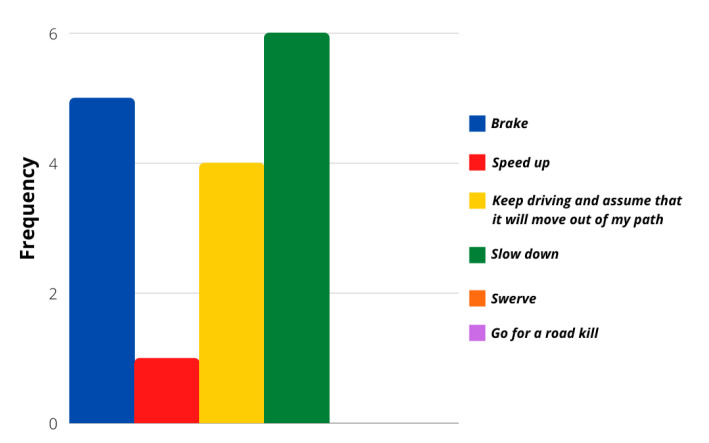
Question 56 results.

**Figure 10:**
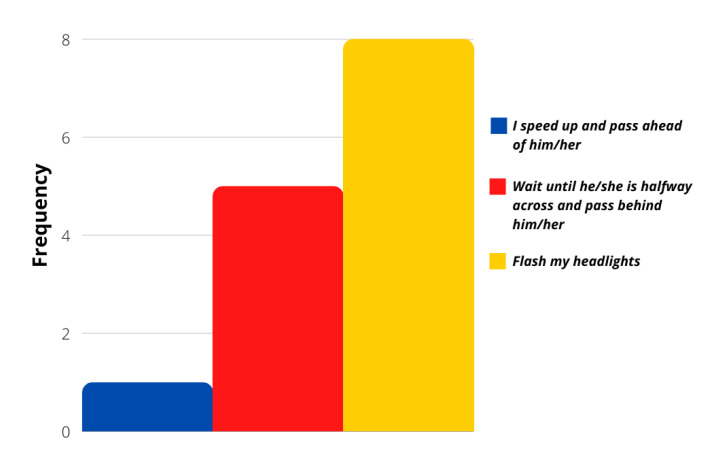
Question 52 results.

**Figure 11:**
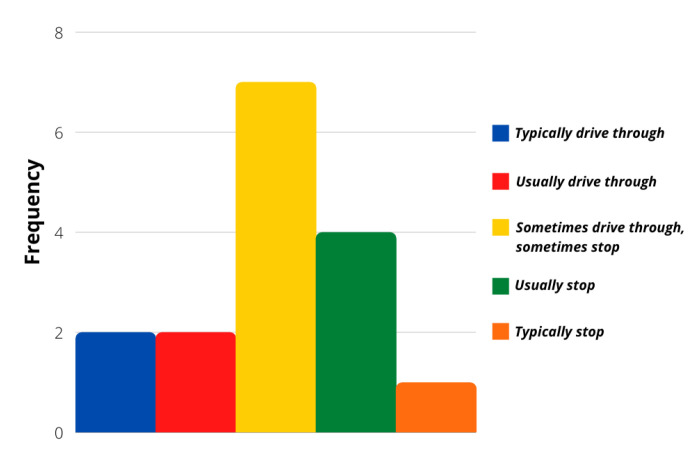
Question 60 results.

The evaluation of more concrete situations involving different stimuli was analyzed with the survey’s last ten questions. Even though most of the answers followed a calm driver personality again, these particular situations showed that a more aggressive reaction frequency was possible. Figure 6, Figure 7, Figure 8 and Figure 9 show that most users would act calmly in stressful situations. However, there is a more significant population that would react irresponsibly or disrespectfully. Also, Figure 10 and Figure 11 show a downright aggressive response from the population since a reckless and disrespectful decision was chosen when evaluating their reactions with pedestrians and cases that required to stop the car instead of keeping in movement.

### 5.2. SDT Results

After evaluating each of the survey questions individually, the next step corresponded to the SDT analysis. Based on the results obtained from the SDT analysis, most of the results were acceptable. Results such as that of persons 1, 9, and 11 obtained the smallest $d^{\prime }$ value, concluding that these people did not detect the objectives of the questions. On the other hand, the people interviewed 3, 5, and 10 obtained the highest value. This result means that the distance between the noise signal and the signal distribution is large. It is sought a large $d^{\prime }$ value since it represents how easy it was to find the stimulus in each question.

Also, this study reveals how much uncertainty the test answers have. Based on this, it is sought that the β value is as small as possible, as is the case of people 4 and 7. Otherwise, people that answered with more significant uncertainty were 2, 5, and 10. The expert results obtained a value of $d^{\prime }=1.46523$, which was higher than 81% of the population. At the same time, the value of β=0.34183 was obtained, which was the smallest value of all population. These results are expected from an expert, with a larger value of $d^{\prime }$ and a smaller value of β than any other user. Table 3 shows the overall results of the SDT analysis of every subject. The subject column is marked on italics with the sole purpose of differentiating the number of subject and their corresponding SDT results.

### 5.3. Driving Personality Test Results

After obtaining the test results of the 11 surveyed persons, 2 driver profiles were obtained: the calm driver and an aggressive driver. When analyzing the survey questions, it was possible to appreciate that a large number of the questions describe the aggressive behavior of the people, while a smaller number of questions qualify the calm behavior of the people. There are questions that were considered not necessary and, therefore, were omitted since they did not influence the two types of behavior that the test showed. On the other hand, of the 11 respondents, the vast majority had a calmer driver profile.

**Figure 12:**
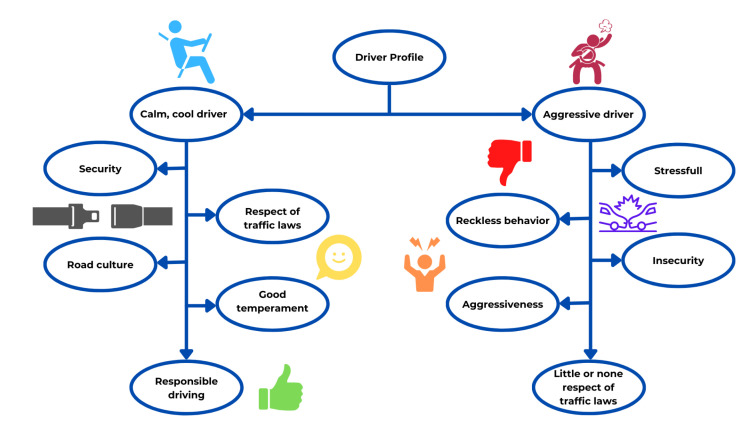
Cognitive map.

To obtain the first approach regarding the driver profiles, a cognitive map (see Figure 12) based on the questionnaire results is used to organize the ideas about the two driver profiles analyzed.

## 6. Discussion

The comparison of the personal treats of the driver with the particular driving laws of a particular place makes it possible to classify the driver into aggressive or passive behavior. This classification allows evaluating under which circumstances that person is putting himself under a possible car accident. The general results of this proposal show that it is possible to classify a qualitative circumstance, like yelling at another driver or letting pass a pedestrian, into a quantitative value that helps the car react to preventing any accident.

However, to implement this kind of analysis into an actual car prototype, it is necessary to evaluate a more significant population of drivers and a bigger population of experts that could determine an average score for determining aggressive or passive driving behaviors. Another limitation of this proposal is that it only evaluates the driver’s conduct; nevertheless, it is also essential to evaluate the particular situation in the driver’s environment at that time. Under very few circumstances, it could be possible that generally aggressive behavior is a passive response that may prevent an accident from happening, being it fault of another driver, or even a technical situation, like the traffic lights not working. In other words, it is still necessary to broaden the evaluation perspectives to create a more robust classification metric.

Finally, it is also important to make sure the driver knows about the car collecting its current personal data while driving, as well as the possible situation of sending that data to a third person, being it a medical staff in charge of the possible accident, or a driver’s contact to inform about that specific situation. It is crucial to determine how the automatic security system, such as the seatbelt tension increase, affects the user when dealing with this type of information. According to Günthner and Proff [37], a properly implemented security system needs to consider the social norm, acceptance, perceived usefulness, perceived ease-of-use, and trust in technology of the user’s treats to prevent undesired outcomes, like a panic attack.

## 7. Conclusions

An inappropriate driving behavior may end in accidents that compromise the driver’s and other involved people’s lives. With the development of intelligent vehicles, it is possible to determine when a person is driving dangerously and prevent a possible accident. Even though there were several psychological, moral, and physical evaluations of a driver’s behavior to determine the presence of aggressive driving conduct, it is still necessary to combine such evaluations into a practical, direct control of the vehicle to effectively prevent an accident.

The main objective of this project was to evaluate the driver personalities of a Mexican population based on a driving situation survey. Signal Detection Theory (SDT) was implemented to determine the user’s ability to determine the best course of action. The results show that most of the time, the users develop a calm attitude towards stressful driving situations. However, the higher β values of the SDT analysis show that most of them were not so sure about selecting the best answers in the survey. However, even though not being sure about their answers, the participants could answer the survey under a calm driver personality.

This implementation allows to combine a qualitative analysis, such as the driving evaluation, into a quantitative control model through SDT to determine if the driver is moving aggressively or not while controlling the car’s seatbelt and velocity actually to prevent, or at least diminish, a genuine accident. The proposed system can detect aggressive driving behavior so that it can be reported and send a warning to health specialists. Besides, in the future, intelligent cars could automatically detect particular health problems according to driving behavior.

For future work, the possibility of using Fuzzy Logic Systems of type 1 and 2 could improve the SDT analysis of the survey answers, decreasing the bias of the uncertainty when answering each of the questions, as shown in [38,39]. Otherwise, classifying the driver’s personality into calm and aggressive could help improve and develop customized collision alert systems that select particular signals and actions depending on the driver’s tendencies.

## Figures and Tables

**Table 1:** Driving personality survey.

1	When drivers indicate they want to pass ahead of me into my lane, I let them through
2	I drive at or below the speed limit.
3	When a driver allows me to pass ahead of him/her, I signal my thanks (smile, wave my hand, etc.)
4	I reduce my speed when I am in a school zone.
5	On rainy/slushy days, I slow down when driving near sidewalks to avoid splashing pedestrians.
6	I talk on the phone while driving.
7	I give other drivers the right-of-way.
8	If I know I have had too much to drink, I find an alternative mode of transportation to get home.
9	I use my signal flashers to let drivers behind me know when I am turning or changing lanes.
10	Even if it is late at night and no other cars are on the road, I still stop at traffic lights or signs.
11	If I am driving slower than other drivers, I refrain from using the left lane.
12	If my passenger(s) try to pressure me to drive faster, I ignore them.
13	Even when weather conditions at night are clear, I still drive with my high beams on.
14	When driving, I insist that all my passengers put their seatbelts on.
15	When waiting to turn from a one-way street, I leave enough space (if possible) so that drivers behind me that are going straight can pass.
16	I check my blind spot before changing lanes.
17	My driving attitude and style change depending on whether a police officer is nearby.
18	At an intersection with a four-way stop, the vehicle that arrives first should go first.
19	Even if a road is divided by a cement median, cars on the opposite side must still stop when the signal lights of a school bus are flashing.
20	Reversing on expressway entrances or exits is allowed.
21	The faster you drive, the more braking distance you will require to come to a full stop.
22	When passing (from the left lane), you are not permitted to pass more than one vehicle at a time.
23	If two or more vehicles arrive at a four-way stop simultaneously, the one on the right should yield to the one on the left.
24	Drivers must stop a minimum of 5 m from a school bus (with its signal lights flashing).
25	When more than one lane is designated for turning, the safest turning method would be to use the left lane.
26	If the beams blind you from an oncoming car’s headlights, you should direct your eyes:
27	Hydroplaning occurs when:
28	As your driving speed increases, your field of vision:
29	In which of the following conditions is the road the MOST slippery?
30	Purposely cutting someone off.
31	Swearing or “flipping the bird.”
32	Tailgating.
33	Honking to make someone drive faster.
34	Zigzagging through traffic.
35	Exceeding the speed limit by 20 mph (30km/h) on city streets.
36	Verbalizing or wishing physical harm to other drivers.
37	Braking suddenly to scare a tailgater.
38	Excessively honking.
39	Flashing your lights in frustration.
40	Trying to run someone off the road.
41	Purposely hitting another driver (with your car or an object)
42	Purposely cutting a driver off to splash water in their car.
43	Stopping your car in anger or insisting that a driver pull over to confront him/her (i.e., because she/he cut you off, stole your parking space, etc.)
44	Rolling your window down to yell at another driver.
45	Chasing another car.
46	It is OK to drive a little recklessly as long as no one is around to get hurt.
47	I think punishment for speeding should be license removal.
48	I wear my seatbelt.
49	I believe there is nothing wrong with breaking a few traffic regulations as long as you have good reflexes (are an alert driver).
50	You are driving along peacefully when a car full of teenagers zooms up behind you. After tailgating you for a few moments, they then proceed to pass you, pull up in front of you, and drastically decrease their speed. All along, they are taunting, laughing, and pointing. What do you do?
51	You are stuck in a traffic jam on a hot summer day. What do you usually do in this situation?
52	A slow pedestrian is crossing the street you want to turn on. What do you do?
53	You are driving down a road at night when you notice that an oncoming driver does not have his/her headlights on. What do you do?
54	You are driving at the speed limit on a low-traffic country road when a car passes you illegally. Which of the following would you most likely do?
55	You are driving down a narrow street late at night when you accidentally bump into a parked car. The dent is minor but still somewhat noticeable. What is your first instinct?
56	You have had a bad day. You were chastised by your boss in front of your colleagues, you had a rude waitress during lunch, and you got splashed on the way to your car. As you are driving through a quiet neighborhood, you see a little squirrel crossing the street about 30 feet (10 m) ahead of you. What is your first instinct?
57	You are at the mall for some last-minute Christmas shopping. Available parking space is sparse buy you manage to spot a car pulling out. You steer towards and wait for the car to leave, but just as you are about to pull in, another car drives up from the opposite side and takes it from you. Unfortunately, you are not sure whether he/she saw you waiting. What do you do?
58	You are on your way to the theater with your niece and nephew to see the latest Disney movie. An aggressive driver recklessly cuts you off as you are chitchatting, forcing you to slam on the brakes. How would you respond?
59	The light turns green, but the driver ahead of you has not noticed. What do you usually do in this situation?
60	You are approaching an intersection, and the light turns yellow. You can make it through if you accelerate, but you have enough time to stop. What do you do?

**Table 2:** Multiple option answers.

26	Straight ahead	Towards the right side of the pavement		Towards the tip of your hood		Towards the horizon	
27	Your brakes get wet	You slide on black ice		You accelerate too quickly in snowy conditions		Your tires lose traction in deep water	
28	Increases	Decreases	Remains the same, no matter what speed you are driving at				
29	Before it starts to rain	A few minutes after it starts raining		After it has finished raining		Once most of the water has evaporated	
50	Take the first exit to shake them off	Reciprocate with the same behavior		Speed up to shake them off	Pull-over and wait for them to go away	Call the police on the cellphone/note their license plate for later	
51	I call someone on the phone	I sit back and try my best to wait patiently		I catch up on work or homework	I vent my frustration by swearing or honking	I try to find a way to get through the traffic (e.g., drive on the shoulder of the road, or zigzag through the cars)	
52	Speed up and pass ahead of him/her	Wait until he/she is halfway across and pass behind him/her		Wait until he/she is near the sidewalk and then turn		Wait until he/she steps entirely into the sidewalk before turning	
53	Ignore him/her		Honk			Flash my headlights	
54	Honk and/or flash my lights	Reciprocate and/or get behind the car and start tailgating		Ignore it and keep driving	Give the driver a dirty look and keep driving		Try to drive the car off the road to teach the driver a lesson
55	Keep driving; the damage is not that bad	Stop to make sure the damage is not too bad, then drive away		Start ringing some doorbells to find the owner of the car		Leave a note on the windshield with your name and number	
56	Brake	Speed up	Go for a roadkill	Slow down	Swerve	Keep driving and assume that it will move out of my path	
57	Look for another space	Get out of the car and peacefully confront the driver	Roll down my window and start screaming at the driver		Get out of the car and angrily confront the driver		Shake my head in anger and find another spot
58	I would ignore the incident and keep driving	I would shake my head in disapproval	I would mutter swear words under my breath	I would shout obscenities at the driver that would make a sailor blush		I would chase after the driver to tell him/her off	I would chase after the driver and coy him/her off in return
59	I wait until he/she notices	I wait for other cars to honk	I wait a few seconds to see if he/she notices, then honk lightly	I honk immediately but lightly	I honk heavily until he/she moves	I honk heavily until he/she moves, then give him/her a dirty look	I try to pass around him/her making sure to give him/her a dirty look before speeding up
60	Typically drive-through	Usually, drive-through		Usually, stop	Typically stop	Sometimes drive through, sometimes stop	

**Table 3:** SDT results.

Subject	H	M	FA	CR	HR	FAR	$d^{\prime }$	β
*Expert*	13	1	14	14	0.92857	0.50000	1.46523	0.34183
*1*	11	3	16	12	0.78571	0.57143	0.61163	0.74294
*2*	11	3	22	6	0.78571	0.78571	0.00000	1.00000
*3*	12	2	6	22	0.85714	0.21429	1.85921	0.77375
*4*	13.5	0.5	24	4	0.96429	0.85714	0.73517	0.34816
*5*	9	5	3	25	0.64286	0.10714	1.60797	2.02203
*6*	11	3	15	13	0.78571	0.53571	0.70200	0.73394
*7*	13.5	0.5	24	4	0.96429	0.85714	0.73517	0.34816
*8*	3	11	7	21	0.21429	0.25000	0.00000	0.91771
*9*	12	2	23	5	0.85714	0.82143	0.14675	0.86425
*10*	9	5	5	23	0.64286	0.17857	1.28693	1.42895
*11*	11	3	17	11	0.78571	0.60714	0.51976	0.75852

## Data Availability

Due the personal data collected through this research and the exclusivity of it, it is not possible to share the questionnaire’s results.
